# Role of TFEB in Autophagy and the Pathogenesis of Liver Diseases

**DOI:** 10.3390/biom12050672

**Published:** 2022-05-06

**Authors:** Shengmin Yan

**Affiliations:** Department of Pathology and Laboratory Medicine, Tulane University School of Medicine, New Orleans, LA 70112, USA; syan2@tulane.edu; Tel.: +1-504-988-0286; Fax: +1-504-988-7389

**Keywords:** TFEB, autophagy, metabolism, fatty liver disease

## Abstract

The transcription factor EB (TFEB) is a master regulator of lysosomal function and autophagy. Mechanistic target of rapamycin (mTOR)-mediated phosphorylation on TFEB is known to regulate TFEB subcellular localization and activity at the lysosomal surface. Recent studies have shown that TFEB also plays a critical role in physiological processes such as lipid metabolism, and dysfunction of TFEB has been observed in the pathogenesis of several diseases. Owing to its ability to improve disease status in murine models, TFEB has attracted attention as a therapeutic target for diseases. In this review, we will present the regulation of TFEB and its role in the pathogenesis of liver diseases, particularly non-alcoholic fatty liver disease (NAFLD).

## 1. Introduction

The transcription factor EB (TFEB) is a master regulator of lysosomal function and autophagy and a member of the microphthalmia family of basic helix-loop-helix leucine zipper transcription factors [1]. Mechanistic target of rapamycin (mTOR)-mediated phosphorylation on TFEB is known to regulate TFEB subcellular localization and activity at the lysosomal surface [1]. As a major transcription regulator of the autophagy-lysosomal pathway, TFEB positively regulates the expression of autophagy and lysosomal biogenesis-related genes, thereby promoting autophagosome formation, autophagosome-lysosome fusion, and the degradation of autophagy substrates [2]. In addition to its roles in autophagy-lysosomal pathway transcriptional regulation, TFEB also plays a critical role in physiological processes such as lipid metabolism [1,3]. A gene network regulated by TFEB has been studied using microarray, chromatin immunoprecipitation sequencing (ChIP-seq), and RNA sequencing in cell lines [4,5,6,7]. TFEB-dependent transcriptome changes in the liver have also been analyzed by microarray in mouse livers overexpressing TFEB [8]. These studies provide invaluable data for understanding TFEB downstream genes. Dysfunction of TFEB has been observed in the pathogenesis of several diseases, including neurodegenerative disease [9,10,11], aging [12], kidney diseases [13,14,15], pancreatitis [16,17], *Salmonella typhimurium* infection [18], and melanoma [19]. Owing to its ability to improve disease status in murine models, TFEB has attracted attention as a therapeutic target for diseases. In this review, we will present the regulation of TFEB and its role in the pathogenesis of liver disease, particularly non-alcoholic fatty liver disease (NAFLD).

## 2. TFEB and Its Regulation

### 2.1. Transcriptional Regulation

The biological functions of TFEB are strictly regulated through transcriptional regulation, post-translational modifications, protein–protein interactions, and spatial organization [1,20,21]. Several transcriptional factors have been found to regulate TFEB expression. The activation of peroxisome proliferator-activated receptor-α (PPAR-α) by its agonist, gemfibrozil, can enhance TFEB activity in brain cells [22]. The same study also observed the recruitment of retinoid X receptor-α (RXR-α), PPAR-α, and peroxisome proliferator-activated receptor gamma coactivator 1-α (PGC1-α) on the PPAR-binding site on the *Tfeb* promoter by reporter assay and chromatin immunoprecipitation studies. The cAMP-responsive element-binding protein hepatic-specific (CREBH) is an endoplasmic reticulum (ER)-tethered, stress-sensing transcription factor. One study shows that CREBH can regulate and interact with PPAR-α and PGC1-α to synergistically induce expression of TFEB upon nutrient starvation [23]. Scavenger receptor class B type I (SCARB1) was shown to regulate TFEB expression by enhancing PPAR-α activation [24]. These studies suggest that PPAR-α is a central transcriptional factor for regulating TFEB expression, particularly under metabolic stress.

Transcriptional factors other than PPAR-α have also been found to regulate TFEB expression. Programmed cell death 4 (PDCD4), a tumor suppressor, suppresses TFEB translation in a eukaryotic initiation factor 4A-dependent manner [25]. Tumor protein P53 activation by mild stress is shown to induce F-box protein 22 expression, which in turn causes the degradation of a transcription suppressor complex containing MYC proto-oncogene (MYC), lysine-specific demethylase 4B (KDM4B), and nuclear receptor corepressor 1 (NCoR1), thereby enhancing transcriptional induction of TFEB [26]. Interestingly, MYC itself also can suppress TFEB expression by directly binding to the promoter of TFEB, which can be abated by the inhibition of histone deacetylases [27]. Spliced X-box binding protein 1 (sXBP1) is a key transcription factor that promotes the adaptive unfolded protein response that has been shown to regulate genes involved in lysosomal function in the liver under fasting conditions [28]. Mechanically, sXBP1 could occupy the −743 to −523 site of the promoter of *Tfeb* and induce TFEB expression.

### 2.2. Post-Transcriptional Regulation

**Figure 1:**
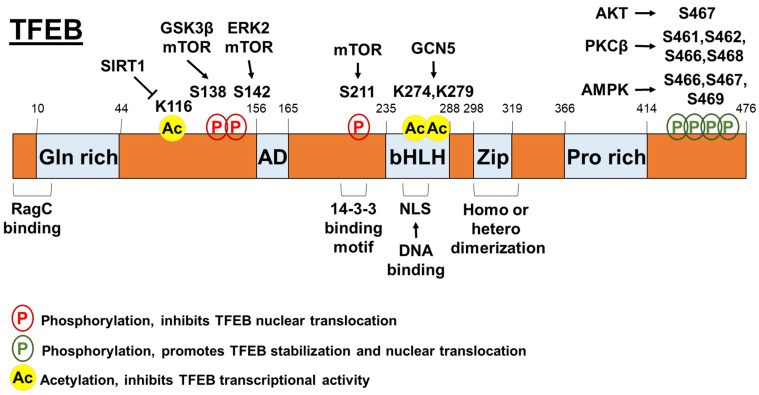
The post-transcriptional regulation mechanisms of TFEB.

TFEB nuclear translocation is highly regulated by its phosphorylation (Figure 1). mTOR is critical for the coordination of cell growth and metabolism [29] and its role in regulating TFEB activity has been well defined. Briefly, mTOR complex 1 (mTORC1) phosphorylates TFEB on Ser211 and triggers the binding of 14-3-3 proteins to TFEB, thereby causing retention of TFEB in the cytosol [30,31,32]. This pathway is regulated by Rag guanosine triphosphatases (GTPases), which can both activate mTORC1 by sensing lysosomal amino acids and determine the localization of mTORC1 and TFEB on the cytosolic surface of lysosomes [33,34]. Indeed, both Rag GTPase-mediated mTORC1-TFEB interaction and active RagC/D heterodimer are required for TFEB phosphorylation [35]. Intriguingly, TFEB can induce RagC/D expression. The overactivation of RagC/D contributes to the kidney phenotype and mTORC1 hyperactivity in folliculin (a RagC and RagD activator) knockout mice. mTOR is also responsible for TFEB nuclear export. Napolitano et al. [36] showed that the subcellular distribution of TFEB is dynamically regulated by its continuous shuttling between the cytosol and the nucleus. This nuclear export seems to be a limiting step of TFEB shuttling, which is mediated by chromosomal maintenance 1 and a mTOR-dependent phosphorylation on S142 and S138 of TFEB.

The phosphorylation of TFEB can be suppressed by signaling pathways other than mTOR [20,21]. For example, TFEB activity can be suppressed by extracellular signal-regulated kinase 2 (ERK2) through the phosphorylation of TFEB at S142 [4]. Interestingly, ERK activation by the serine-threonine kinase RIP1 negatively regulates TFEB activity and modulates basal autophagic flux, suggesting a crosstalk between cell death pathway and autophagy pathway [37]. Mitogen-activated protein kinase kinase kinase kinase 3 (MAP3K3) can phosphorylate TFEB in an amino acid-dependent manner, which is required for TFEB interaction with mTORC1-Rag GTPase-Ragulator complex and TFEB cytosolic sequestration [38]. Interestingly, AKT also can directly suppress TFEB nuclear translocation by phosphorylating TFEB at S467, independently of mTORC1 [39]. Some signaling pathways have also been shown to activate TFEB. Phosphorylation of TFEB at S461, S462, S466, and S468 by protein kinase C β (PKCβ) can stabilize and increase the activity of TFEB [40]. AMP-activated protein kinase (AMPK)-dependent phosphorylation of TFEB at S466, S467, and S469 is required for the transcriptional activity of TFEB [41]. Additionally, AMPK-mediated signaling can increase levels of coactivator-associated arginine methyltransferase 1 (CARM1), which serves as a transcriptional coactivator through TFEB [42].

In addition to the phosphorylation of TFEB, protein acetylation is also important for regulating TFEB activity (Figure 1). One study showed that general control non-repressed protein 5 (GCN5) induced TFEB acetylation at K274 and K279, and decreased the transcriptional activity of TFEB by inhibiting its dimerization and its capability to bind the promoter regions of target genes [43]. In microglia, deacetylase sirtuin-1 (SIRT1) can bind and deacetylate TFEB at K116, thereby enhancing TFEB transcriptional function [44]. Interestingly, a well-established histone deacetylase inhibitor, suberoylanilide hydroxamic acid, can activate lysosomal function in human cancer cells by enhancing TFEB acetylation at K91, K103, K116, and K430 [45]. Besides the acetylation/deacetylation process, the protein turnover of TFEB also alters TFEB activity. A chaperone-dependent E3 ubiquitin ligase, STIP1 homology and U-Box containing protein 1 (STUB1) can preferentially target inactive phosphorylated TFEB for degradation by the ubiquitin-proteasome pathway, thereby increasing TFEB activity [46].

### 2.3. Calcium Signaling

Lysosomal calcium signaling plays a critical role in regulating TFEB activity [47]. The activity of phosphatase calcineurin is regulated by lysosomal calcium release through mucolipin 1 (MCOLN1) under stressed conditions. Calcineurin can bind and dephosphorylate TFEB, thereby promoting its nuclear translocation [48]. Evidence has shown that calcium signaling-mediated TFEB activation is involved in multiple biological processes. Coxsackievirus B3 (CVB3) can cause viral myocarditis and neurological disorders in infants and young children [49]. The CVB3 virus-encoded proteinases may cause autophagy dysfunction by inducing calcineurin-dependent TFEB nuclear translocation. Interestingly, CVB3 proteinase 3 C also participates in the proteolytic processing of TFEB and attenuates its transcriptional activity. A P38 inhibitor, SB202190, can promote TFEB nuclear translocation and subsequently enhance autophagy and lysosomal biogenesis in a manner dependent on ER calcium-related calcineurin activation [50]. Fibroblast growth factor 21 (FGF21), a fasting-induced hormone, can mobilize calcium from the ER and activate the transcriptional repressor downstream regulatory element antagonist modulator, thereby inhibiting the expression of E3 ligase midline-1. The inhibition of midline-1 causes the accumulation of protein phosphatase PP2A, which can dephosphorylate and activate TFEB [51]. Interestingly, TFEB can also activate calcium channel MCOLN1 and raise intracellular calcium levels, thereby promoting the fusion between lysosomes and plasma membrane and regulating lysosomal exocytosis [52]. Overall, these studies suggest that cellular calcium signaling plays a critical role in regulating TFEB activity.

### 2.4. Other Regulation Mechanisms

Liquid-liquid phase separation can compartmentalize transcriptional condensates for gene expression and has been shown to be a critical mechanism for the transcriptional regulation of gene expression [53]. TFEB can form distinct puncta that colocalize with the mediator complex and with mRNAs of its target genes [54]. Intriguingly, inositol polyphosphate multikinase can inhibit liquid-liquid phase separation of TFEB and dissolve TFEB condensates, thereby negatively regulating autophagy activity. MircroRNAs (miRNAs) can also regulate TFEB transcriptional activity. MiR-30b-5p suppresses the transcriptional activity of TFEB by translocating into the nucleus and binding to the coordinated lysosomal expression and regulation elements that are required for dephosphorylated TFEB to recognize and induce expression of its downstream genes [55].

## 3. TFEB and Autophagy

Autophagy, from the Greek auto (self) and phagein (to eat), is an evolutionally conserved degradation process that delivers cytoplasmic cargo (macromolecules or organelles) to the lysosome [56]. Autophagy is critical for maintaining biological homeostasis and its dysfunction contributes to the pathogenesis of various diseases, including tissue injury, microbial infection, tumorigenesis, neurodegeneration, and aging [56]. Macroautophagy, microautophagy, and chaperone-mediated autophagy (CMA) are three major types of autophagy that have been identified and frequently studied [57]. The most well-defined autophagic process, the macroautophagic process, includes three key steps: (1) the sequestration of cytosolic materials into autophagosomes, (2) the transportation of autophagosomes to the lysosome, and (3) the formation and degradation of autolysosomes [57]. Microautophagy is mainly studied in yeast but can also be observed in mammalian cells. The microautophagic process refers to a direct engulfment of cytoplasmic cargo at the limiting membrane of the lysosome, thereby mediating both invagination and vesicle scission into the lumen of lysosomes [57]. CMA is mediated by chaperones such as the heat shock-cognate protein of 70 kDa, and specific protein targets are shuttled via the chaperones across the lysosomal membrane for degradation in the lumen [57].

Among these three types of autophagic process, macroautophagy (hereafter simply autophagy), is the most active form and is modulated by various signaling pathways at different biological levels [56,58,59]. As a master regulator of lysosomal activity, the role of TFEB in regulating autophagy has been extensively studied [4,30,31,33]. Interestingly, TFEB activity can also be altered by several key factors using autophagy machinery. The kinase PTEN-induced kinase 1 (PINK1) and ubiquitin ligase Parkin are critical for the selective elimination of damaged mitochondria through autophagy (i.e., mitophagy) [60]. Nezich et al. [60] have shown that nuclear translocation of TFEB and its transcriptional activity are dependent on PINK and Parkin during mitophagy. Parkin-mediated TFEB translocation also requires autophagy-related gene (ATG) 9A and ATG5 activity, and the activation of Rag GTPases prevents TFEB translocation during mitophagy. The lipidation of microtubule-associated protein 1A/1B-light chain 3 (LC3) is a key step in the autophagic process. Nakamura et al. [61] found that lysosomal damage can recruit LC3 on lysosomes, where the lipidated LC3 facilitates calcium efflux by interacting with the lysosomal calcium channel MCOLN1, thus causing TFEB activation. Sequestosome 1 (P62/SQSTM1) is a protein considered as a substrate for autophagy. Pan et al. [62] showed that systemic proteasome inhibition increases P62 levels and induces myocardial autophagy. Mechanically, a proteasomal malfunction-induced MOCLN1-calcineurin-TFEB-P62 pathway contributes to the induction of autophagy. Interestingly, P62 may also exert a feed-forward effect on TFEB activation, suggesting that TFEB can be a central factor that links the ubiquitin-proteasome system to the autophagic-lysosomal pathway.

Given its master role in regulating lysosomal homeostasis and autophagy, TFEB undoubtedly contributes to various pathophysiological changes. For example, Pastore et al. [63] have shown that TFEB and TFE3 display a circadian activation over a 24-h cycle in mice. Genetic deletion of TFEB and TFE3 causes dysregulation of autophagy over the diurnal cycle and alters gene expression, leading to abnormal circadian wheel-running behavior. Enhancing TFEB-mediated autophagy can also improve neurodegenerative changes in mice. Decressac et al. [64] have shown that excess cellular levels of alpha-synuclein in nigral dopamine neurons are associated with a decline in markers of lysosome function and a cytoplasmic retention of TFEB in a rat model of alpha-synuclein toxicity. Overexpression of TFEB reverses the changes in lysosomal function in this rat model, providing robust neuroprotection via the clearance of alpha-synuclein oligomers. Indeed, trehalose, a natural disaccharide and TFEB activator, has been shown to promote autophagy by activating TFEB and ameliorating disease phenotypes in multiple neurodegenerative disease models [65,66].

## 4. TFEB and Liver Disease

**Figure 2:**
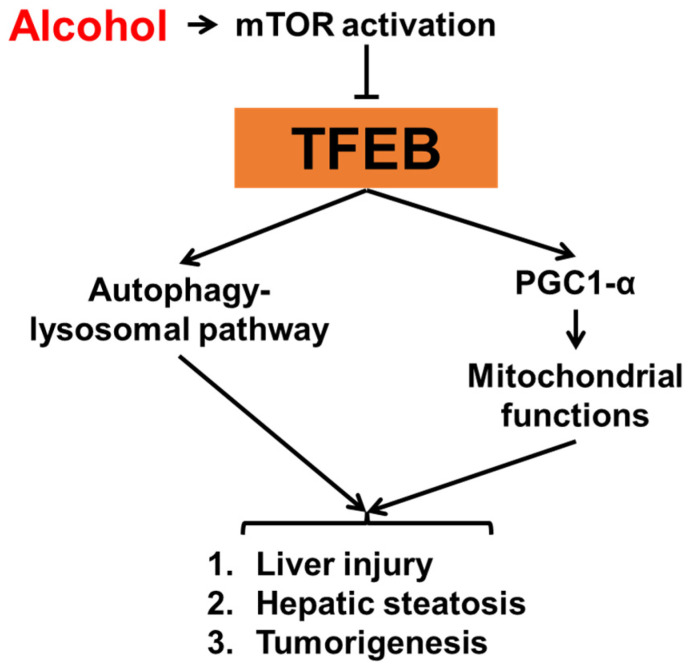
The role of TFEB in alcohol-associated liver injury.

Autophagy is critical for liver homeostasis [67,68,69]. Roles of autophagy in the pathogenesis of alcohol-associated liver diseases (ALD) have been well characterized. Studies from murine models have shown that acute alcohol treatment induces autophagy [70], whereas chronic alcohol treatment suppresses autophagy in the liver [71]. Despite different impacts of alcohol on autophagy by different treatment schemes, the activation of autophagy improves alcohol-induced liver injury while the inhibition of autophagy enhances it. Consistent with the autophagy status, the nuclear content of TFEB is increased in mouse livers following acute alcohol administration but decreased following chronic alcohol treatment [72]. The role of TFEB in the pathogenesis of ALD has been extensively elucidated in a study by Chao et al. (Figure 2) [73], in which hepatic levels of TFEB protein were analyzed in livers from different murine ALD models. Interestingly, TFEB proteins were decreased in both total lysates and nuclear fractions when mice were given either chronic-plus-binge or a long-term chronic alcohol treatment, whereas neither a short-term chronic nor an acute gavage alcohol treatment caused TFEB alteration. In patients with alcoholic hepatitis, the authors also observed a decrease in nuclear contents of TFEB in the liver, indicating that TFEB activity may contribute to the pathogenesis of ALD in both humans and murine models. Further experimental evidence shows that an overexpression of TFEB improves, while a decrease in TFEB enhances alcohol-induced liver injury in mice, following chronic-plus-binge alcohol treatment. This effect of TFEB seems to be related to mTOR activation. In addition to mouse models, TFEB is also altered and involved in alcohol-induced hepatic steatosis in male Wistar rats given an alcohol liquid diet for six weeks [74]. Interestingly, this study also shows that withdrawal of alcohol can restore nuclear TFEB contents and thereby reverse hepatic steatosis. However, despite the strong evidence that TFEB is impaired in ALD, the TFEB activator trehalose unexpectedly failed to improve alcohol-impaired TFEB and liver injury in mice, which keeps the translational potential of targeting TFEB for ALD uncertain [75].

Roles for TFEB-mediated autophagy have also been shown in other types of liver injury. Deficiency of alpha-1 antitrypsin (AAT) leads to polymerization and aggregation of mutant AAT, causing liver injury [76]. Interestingly, TFEB-induced autophagy decreases toxic mutant AAT polymer and improves liver pathology in a murine model of AAT deficiency [77]. Diclofenac, a nonsteroidal anti-inflammatory drug, can inhibit autophagic flux in hepatocytes [78]. Transfection of TFEB has been shown to restore lysosomal pH and thus autophagic flux in diclofenac-induced hepatocyte damage. Activation of TFEB by carbon monoxide can protect lipopolysaccharide/D-galactosamine-induced liver injury in mice [79]. Taken together, this evidence suggests that dysfunction of TFEB activity involves in different types of liver damage, and targeting TFEB as a therapy seems promising.

Emerging evidence has shown the contribution of TFEB in the development of cancers. TFEB affects cancer progression mainly through its functions in lysosome homeostasis, metabolism, cell cycle regulation, and epithelial-mesenchymal transition [20]. Although genetic alterations of TFEB are involved in the development of tumors in kidney, exocrine pancreas, and melanomas [20], alterations and functions of TFEB in liver cancer have not yet been fully identified. One recently published study has shown the role of TFEB in controlling liver cell fate during development and regeneration, which may also contribute to the development of biliary cancer [80]. It also suggested that the expression of TFEB is enriched in ductal/progenitor cells and contributes to murine liver cell fate during development and regeneration by direct transcriptional regulation of SRY-box transcription factor 9 (SOX9). Overexpression of TFEB in either hepatocytes or cholangiocytes can cause biliary cancer after DDC-diet-induced liver injury by increasing the number of progenitor/cholangiocyte-like cells. Interestingly, liver-specific TFEB knockout mice seem to have fewer larger tumors in an HCC model with a combination of diethylnitrosamine (DEN) and chronic ethanol-feeding treatment [81], suggesting that the role of TFEB in liver tumorigenesis may vary from different etiologies.

## 5. TFEB and NAFLD

### 5.1. TFEB and Metabolism

**Figure 3:**
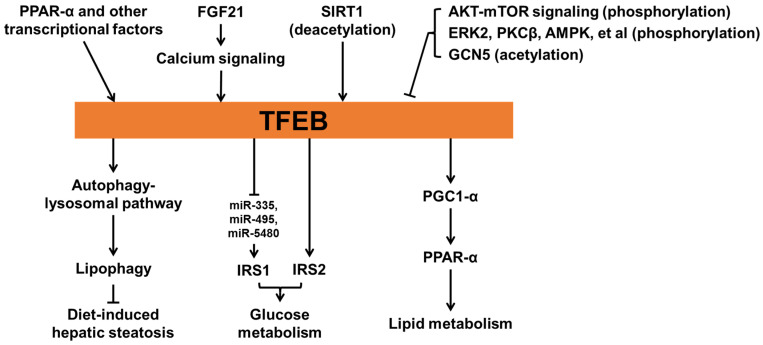
The role of TFEB in metabolic homeostasis.

Evidence has shown that TFEB is critical for maintaining metabolic homeostasis (Figure 3) [3,82]. Fasting-induced FGF21 signaling can activate Jumonji-D3 histone demethylase (JMJD3) in mice, thereby epigenetically upregulating global autophagy network genes, including *Tfeb* [83]. Fasting-promoted expression of the TFEB orthologue HLH-30 is also observed in *C. elegans* [84], suggesting a conserved effect of fasting on the expression of TFEB in both mice and *C. elegans*. The function of fasting-induced TFEB expression has been elucidated. In this study, Settembre et al. [8] found that fasting-induced TFEB expression regulates lipid metabolism. TFEB expression is increased in livers, kidneys, and muscles following fasting in mice. This increase in TFEB is also found in MEF cells, hepatocytes, and *Caenorhabditis elegans* following starvation but drops significantly after refeeding, indicating that TFEB expression is regulated by nutrient status. Mechanically, TFEB directly mediates the expression of PGC1-α and thus controls the activity of PPAR-α, thereby contributing to lipid metabolism. The study also showed that TFEB-mediated lipid breakdown requires autophagy. TFEB-induced PGC1-α expression is also found in adipocytes and provides beneficial effects on diet-induced metabolic dysfunction.

The roles of TFEB in overnutrition status have also been characterized. Evans et al. [85] found that mice with adipocyte-specific TFEB overexpression are protected from diet-induced metabolic dysfunction, mainly due to increased metabolic rate. Mechanistic studies suggest that overexpression of TFEB promotes adipocyte browning through PGC1-α. Li et al. [86] showed that TFEB with phosphorylation on S142 can be further phosphorylated at S138 by glycogen synthase kinase 3β (GSK3β), an enzyme that is essential for glucose homeostasis. The phosphorylation of both sites, but not either alone, can cause nuclear export signal and lead TFEB to be re-exported to cytoplasm. Thus, the alteration of TFEB by GSK3β and the critical roles of AKT-mTOR signaling in both amino acid and glucose homeostasis suggest that TFEB can be controlled by the availability of both glucose and amino acid. Evidence from endothelial cell (EC)-specific TFEB knockout or transgenic mice following a high-fat diet (HFD) has shown a role of EC-TFEB in glucose metabolism. In the same study, Sun et al. [87] found that following HFD feeding, EC-specific TFEB transgenic mice exhibited improved glucose tolerance while EC-specific TFEB-knockout impaired it. Mechanically, TFEB can directly upregulate insulin receptor substrate 2 (IRS2) and increase IRS1 protein levels by downregulating miR-335, miR-495, and miR-5480.

Increasing TFEB activity is beneficial to diseases related to metabolic syndrome. EC-specific TFEB transgene can inhibit endothelial cell inflammation and reduce atherosclerosis development in apolipoprotein E knockout mice [88]. Moreover, TFEB-mediated autophagy contributes to mesenchymal stem cell-promoted M2 polarization of macrophages, thereby alleviating diabetic nephropathy [14]. Finally, TFEB also controls metabolic flexibility in muscles during exercise in a manner independent of PGC1-α. Mansueto et al. [89] found that TFEB can translocate into myonuclei during physical activity and mediate the expression changes of genes related to glucose homeostasis, thereby regulating glucose uptake and glycogen content. The same study also found that TFEB can regulate mitochondrial biogenesis and function in muscles.

### 5.2. TFEB in NAFLD/NASH and Its Therapeutic Potential

Obesity is strongly associated with numerous diseases, including heart disease, NAFLD, stroke, type 2 diabetes, and certain types of cancer [90,91]. Many of these conditions lead to preventable, premature death and contribute to the high annual medical cost of obesity in the U.S. [92]. NAFLD is a common obesity-related pathological condition that is intimately associated with the clinical features of metabolic syndrome [93,94,95]. NAFLD is usually characterized by the presence of excessive fat accumulation in the liver without other recognized causes of hepatic lipid accumulation [96]. As a leading cause of chronic liver disease, patients with NAFLD can develop non-alcoholic steatohepatitis (NASH), hepatic fibrosis, and eventually hepatocellular carcinoma (HCC) [96]. Moreover, NAFLD is also a systemic disease that can increase the risk of extra hepatic complications [96,97]. Patients with NAFLD develop increased clinical causes of cardiovascular morbidity and mortality, including atherosclerosis, cardiomyopathy, and arrhythmia [98,99]; however, although the mortality rate from NAFLD is increasing in the U.S., FDA-approved therapies for NAFLD are still lacking [97].

Emerging evidence continues to demonstrate the role of TFEB in the pathogenesis of liver steatosis and NAFLD. Our previous study showed that nuclear contents of TFEB, as well as the phosphorylation levels of ribosomal protein S6 kinase β-1 (S6K), a classic target for mTORC1, are both oscillated in the liver during long-term HFD feeding, indicating that mTORC1 signaling and TFEB activity are dynamically altered by overnutrition [100]. Moreover, the same oscillation is observed in the expression of TFEB downstream genes, including genes-related to lysosomal and autophagic functions. This observation is further confirmed by measuring the activity of lysosomal enzymes and autophagic degradation in the liver, suggesting that the oscillation of mTORC1 and TFEB activity dynamically regulate autophagy following HFD-feeding. The oscillation of lipophagy is also observed in HFD-fed mouse livers. In NAFLD patients, nuclear contents of TFEB are observed and negatively correlated with steatosis score but not body mass index (BMI). Our data suggest that TFEB activity is compromised in fatty livers, which may be related to reduced lipophagy activity. Finally, our study shows that either overexpression of TFEB or suppression of mTORC1 can improve hepatic status of HFD-fed mice, whereas overexpression of a constitutively activated RagA mutant that can support mTORC1 activation without amino acid stimulation impairs liver function. Overall, our study elucidates the critical role of TFEB in hepatic lipid homeostasis and shows that loss of TFEB function contributes to the pathogenesis of diet-induced fatty liver.

TFEB also plays a role in the homeostasis of cholesterol. Wang et al. [101] found that TFEB promotes the gene expression of cytochrome P450 family 7 subfamily A member 1 (*Cyp7a1*), a key gene for bile acid synthesis. TFEB nuclear translocation is activated by cholesterol-induced lysosomal stress, whereas bile acid-induced FGF15/19 inhibits TFEB nuclear translocation by mTOR/ERK signaling and TFEB phosphorylation in the liver. This regulatory loop is critical for hepatic cholesterol and bile acid homeostasis. GSK2330672, an inhibitor of apical sodium-dependent bile acid transporter (ASBT), can cause increased fecal bile acid excretion and reduce enterohepatic levels of bile acids. ASBT inhibition reduces ileal FGF15 expression and increases nuclear TFEB, thereby inducing the expression of TFEB target genes. Furthermore, in mice fed a Western diet, ASBT inhibitor significantly improves hepatic steatosis in a manner correlated with the increase in nuclear TFEB in the liver. Finally, hepatic TFEB overexpression by adenovirus significantly reduces hepatic and plasma levels of cholesterol, while hepatic TFEB knockdown exacerbates hypercholesterolemia in Western diet-fed mice. Taken together, this study pinpoints a key role of TFEB in balancing hepatic bile acid and cholesterol homeostasis via the gut-liver axis, which is likely related to bile acid-mediated intestinal farnesoid x receptor (FXR) activation. Interestingly, another study also shows that FXR can directly suppress TFEB expression at the transcriptional level [102], suggesting that FXR may be another regulator of TFEB function.

Given the critical role of TFEB in hepatic lipid metabolism, accumulating evidence has shown that TFEB can be a promising therapeutic target for metabolic syndrome. Wang et al. [103] identified small-molecule agonists of TFEB using a nanotechnology-enabled high-throughput screen and found three novel compounds that are capable of promoting autophagolysosomal activity. These three compounds include a clinically approved drug, digoxin; a marine-derived natural product, ikarugamycin; and a synthetic compound, alexidine dihydrochloride. Mechanically, these compounds activate TFEB via three distinct calcium-dependent pathways. In murine models, these compounds confer hepatoprotection against diet-induced steatosis. Another compound, MSL, identified by Lim et al. [104], can activate calcineurin and induce TFEB, thereby accelerating intracellular lipid clearance. MSL treatment also improves the metabolic profiles of *ob/ob* mice. TFEB can also be activated by other treatments for hepatic steatosis, including ezetimibe [105], procyanidin B2 [106], formononetin [107], liraglutide [108], fenofibrate [109], and metformin [110]. Overall, evidence strongly suggests that TFEB is a promising therapeutic target for improving hepatic steatosis.

## 6. Conclusions and Perspectives

Despite the unmet clinical need and attractive commercial opportunity, no therapies have been approved by Food and Drug Administration (FDA) for fatty liver disease. Clinical trials for NAFLD/NASH treatment have shown some encouraging evidence with several drug candidates through late-stage clinical development [111]. These drug candidates include anti-fibrotic/inflammatory compounds, FXR agonists, FGF analogs, PPAR modulators, and compounds directly targeting certain metabolic pathways [111]. Interestingly, many of these therapeutic targets have also been shown to regulate TFEB function, including PPARs, FXR, FGF15/19, FGF21, and several kinases. Given the master role of TFEB in regulating lysosomal homeostasis and autophagy, targeting TFEB may provide a unique therapeutic approach.

Taken together, recent studies have expanded the function of TFEB from a master regulator of lysosomal homeostasis and autophagy to a critical contributor of metabolic homeostasis. TFEB’s role in the pathogenesis of liver disease are well known. TFEB is also a promising therapeutic target for liver diseases, particularly fatty liver disease. Future studies on molecular mechanisms and specific agonists of TFEB will help to develop therapeutic approaches for fatty liver disease.

## Abbreviations

AAT	alpha-1 antitrypsin
AMPK	AMP-activated protein kinase
ASBT	Apical sodium-dependent bile acid transporter
ATG	Autophagy-related gene
CARM1	coactivator-associated arginine methyltransferase 1
ChIP-seq	Chromatin immunoprecipitation sequencing
CMA	Chaperone-mediated autophagy
CREBH	The cAMP-responsive element-binding protein, hepatic-specific
CVB3	Coxsackievirus B3
*Cyp7a1*	Cytochrome P450 family 7 subfamily A member 1
DEN	diethylnitrosamine
ER	Endoplasmic reticulum
ERK	Extracellular signal–regulated kinase
FGF	Fibroblast growth factor
GCN5	General control non-repressed protein 5
GSK3β	Glycogen synthase kinase 3β
GTPases	Rag guanosine triphosphatases
IRS	Insulin receptor substrate
JMJD3	Jumonji-D3 histone demethylase
KDM4B	lysine-specific demethylase 4B
LC3	Microtubule-associated protein 1A/1B-light chain 3
MAP3K3	Mitogen-activated protein kinase kinase kinase kinase 3
MCOLN1	Mucolipin 1
mTORC1	mTOR complex 1
MYC	MYC proto-oncogene
NAFLD	Non-alcoholic fatty liver disease
NASH	Non-alcoholic steatohepatitis
NCoR1	Nuclear receptor corepressor 1
P62/SQSTM1	Sequestosome 1
PDCD4	Programmed cell death 4
PGC1-α	Peroxisome proliferator-activated receptor gamma coactivator 1-α
PINK1	PTEN-induced kinase 1
PKCβ	Protein kinase C β
PPAR-α	Peroxisome proliferator-activated receptor-α
RXR-α	Retinoid X receptor-α
SIRT1	Sirtuin-1
S6K	S6 kinase β-1
SOX9	SRY-box transcription factor 9
SCARB1	Scavenger receptor class B type I
STUB1	STIP1 Homology And U-Box Containing Protein 1
sXBP1	Spliced X-box binding protein 1
TFEB	The transcription factor EB
